# Venous malformation differential diagnosis with an odontogenic abscess on a pediatric patient. Case report and review of the literature

**DOI:** 10.4317/jced.61416

**Published:** 2024-03-01

**Authors:** María Álvaro-Martinez, Carolina Cuesta-Urquía, Luis Ortiz-Peces, Jorge Noguera-Tomás, Teresa González-Otero, José- Luis Cebrián-Carretero

**Affiliations:** 1 Department of Oral and Maxillofacial Surgery, Hospital Universitario La Paz. Paseo de la Castellana 261. 28046 Madrid, Spain

## Abstract

Venous malformations (VMs) are aberrant venous vessel angiogenesis present at birth. However, they can become apparent later in life, debuting in early childhood. This poses a clinical quest for surgeons, dentists, and pediatricians, as they might appear as a compressible mass in the head and neck region, not uncommonly mistaking them for odontogenic abscesses or other soft tissue tumors. The differential diagnosis can be challenging and imaging techniques are often needed. Ultrasounds are extremely useful initially as other diagnostic tools can be potentially harmful in the context of a VM. MRI is key as it provides accurate extension and location information, and allows to plan invasive treatment alternatives if the patient requires it. In this article, we present the case of a 6-year-old girl who was treated by mistake for an infection upon the diagnosis of an incipient odontogenic abscess instead of a venous malformation, and a literature review on VMs.

** Key words:**Venous malformation, odontogenic abscess, differential diagnosis.

## Introduction

VMs are low-flow vascular anomalies present at birth but can be diagnosed in later stages in life. They are most frequently found in the head and neck. Within the oral cavity, they are mostly located on the lips (1). Clinically, they may appear as a non-pulsatile mobile mass, not warm and compressible to palpation, allowing differentiation from arteriovenous malformations. On occasions, they present a blue tint of the overlying skin, which makes diagnosis easier (2). However, when this trait is absent, an acute increase in volume poses a clinical challenge, sometimes leading to confussion with an odontogenic abscess. The differential diagnosis of vascular malformations is then a challenge for clinicians. In this article, we describe a case in which a 6-year-old girl was mistakenly treated for an infection upon the diagnosis of an incipient odontogenic abscess, and we provide a literature review on venous malformations.

## Case Report

**Figure 1:**
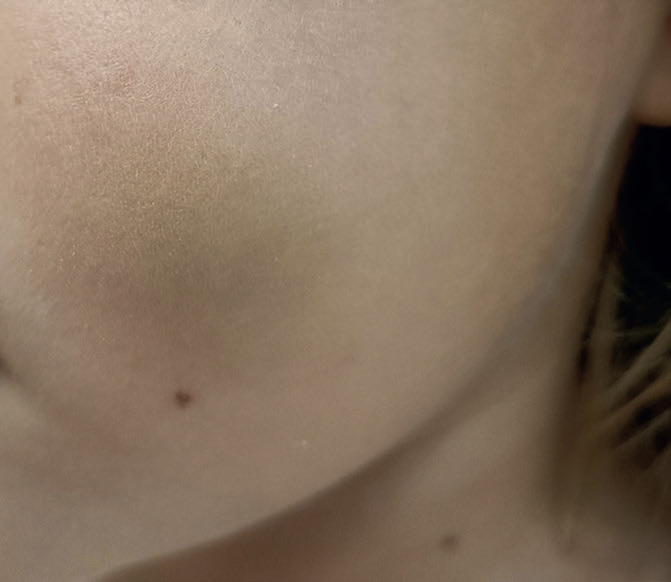
Clinical exploration of our patient upon her arrival to our Hospital. A change of coloration in the left cheek can be observed, presenting a blue tint.

**Figure 2:**
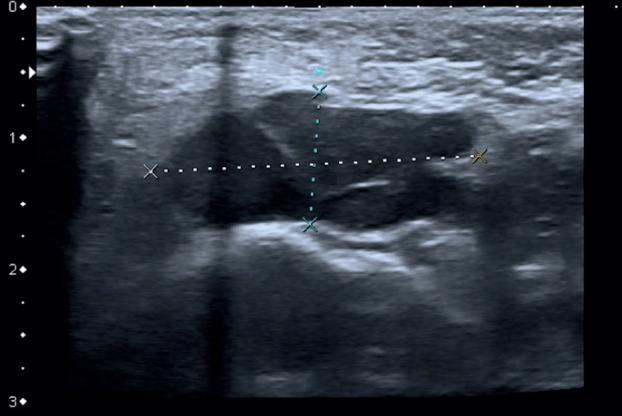
Ultrasound images. Lesion on B mode. Echography of the left cheek: polilobulated hypoechogenic lesion, well defined and embedded in deep layer of subcutaneous tissue anterior to masticatory muscles without infiltration of approximately 1x2x3 cm. It is compressible. On its lateral left side, there are two small vessels with phasic flow suggestive of veins and it does not present flow on Doppler Mode. Taking into account the clinical and radiologic correlate, the lesion is suggestive of a thrombosed venous malformation.

**Figure 3:**
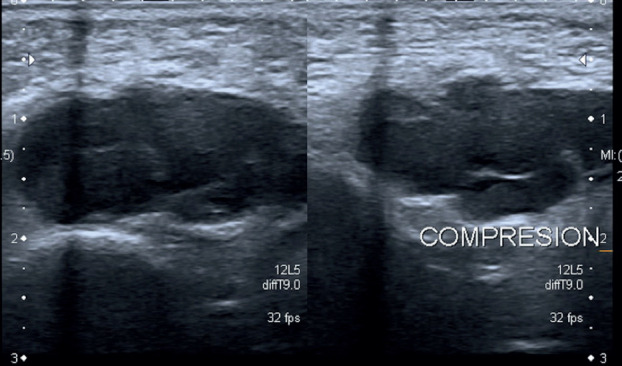
Ultrasound images. Lesion before and after compression; Lesion on doppler Mode. Echography of the left cheek: polilobulated hypoechogenic lesion, well defined and embedded in deep layer of subcutaneous tissue anterior to masticatory muscles without infiltration of approximately 1x2x3 cm. It is compressible. On its lateral left side, there are two small vessels with phasic flow suggestive of veins and it does not present flow on Doppler Mode. Taking into account the clinical and radiologic correlate, the lesion is suggestive of a thrombosed venous malformation.

A 6-year-old girl was referred to our hospital due to a persistent painful mass on the left cheek. She had attended another hospital five days earlier, where she had been diagnosed with an incipient odontogenic abscess. For that reason, she was treated with Amoxicillin and Clavulanic Acid in a suspension of 250/62.5 mg in 5 ml, given 5 ml every 8 hours for 7 days. An ambulatory ultrasound- guided fine needle aspiration was solicited if the lesion did not disappear on the following weeks. On arrival, the patient did not show any signs of improvement, reported worsening pain and referred difficulty to perform a complete oral aperture. During the clinical examination, she presented a mass, well delimited and mobile to palpation. The overlying skin presented a blue tint, which her relatives reported to have appeared only a few hours earlier (Fig. 1). The intraoral exploration revealed no suggestive findings of teeth involvement, with an absence of apparent caries, no odontalgia and no collections or suppuration in any of the intraoral four quadrants. No history of trauma was reported. Based on these findings, the main diagnosis was a soft tissue tumor, of probable vascular origin due to the skin discoloration. Given this suspicion, an echography was requested, which confirmed a thrombosed venous malformation, measuring 1x2,3x3cm, located superficial to the masticatory muscles (Figs. 2,3). The patient was prescribed aspirin, 125 mg/24 hours for three months to resolve the thrombotic event, and was included in the Committee for Congenital Vascular Malformations to decide on an interdisciplinary management. A conservative approach was agreed upon with clinical follow up.

## Discussion

VMs are classified within vascular malformations, according to the latest International Society for the Study of Vascular Anomalies (ISSVA) classification system (3). Venous malformations affect 1,5% of the population, and 2/3 of those cases correspond to VMs (4).

VMs are the most common type of congenital vascular anomaly. The most common location is the head and neck, accounting for up to 40% of the cases. Moreover, they are also the most frequent vascular malformations within the head and neck (5). Within the oral cavity, they account for 6,4% of all the lesions, with the lips and oral mucosa being the most frequent locations (6).

The characteristic clinical exploration of a VM is a compressible mass, mobile to palpation and not adhered to deep layers of the skin, with absence of fluctuation. VMs swell when there is a rise in central venous pressure and they may also infiltrate muscle and bone (7). They tend to grow with age and increase in size during pregnancy, thus upon a mass which exhibits the characteristics presented above in an adolescent or pregnant women, this lesion might be suspected (8). They may discolor the overlying skin with a blue shade if they are embedded in the epidermis, although it is not imperative if they are located in deeper skin layers. The lack of this characteristic can be misleading and it is not infrequent to mistake a VM with another tumor, cyst or infectious process, such as abscesses, for example of odontogenic origin, as happened in the present case. However, with a correct history and physical examination, the diagnosis can be accurate in 90% of the cases (8,2).

Among the most common complications, ulceration, thrombosis and compression of adjacent structures account for a large proportion of the cases. Thrombosis and phlebolith formation can result into pain and swelling. However, the most concerning complication in the head and neck is airway compression. It may cause dyspnea, dysphagia or interference with speech. Another concerning outcome is bleeding, which can lead to life threatening situations depending on the location. It is also important to take into account the aesthetic involvement as a cause of morbidity (9).

As for the imaging techniques, it is useful to ask for an echography, as it is innocuous, provides anatomical information and avoids complications derived from more aggressive procedures, such as biopsies or draining the tumor if other diagnosis are suspected (abscesses, bruises…) Doppler Mode is especially useful for differential diagnosis with arteriovenous malformations and follow up. Moreover, it requires little collaboration from the patient, which provides to be an advantage in the pediatric population (10). On echography, VMs are characterized by being hypoechoic and with low flow. An acoustic shade might be present if there are phleboliths within the venous system. In some cases, patients may benefit from an orthopantomography, which will depend on their age and clinical suspicion. It allows ruling out an odontogenic origin. Once diagnosed, MRI is the gold standard, but provides the inconvenient that requires from the collaboration of the patient, which can sometimes be challenging in children. For that reason, it might be necessary to sedate or anesthetize the patients. Nonetheless, it is often solicited as it provides the most accurate information as to location, extension, information about other organs affected and distinguish it from other diagnosis (1).

There are no defined guidelines for the management of pediatric patients with venous malformations. In children, the indication for an intervention is led by the expected evolution in the long term of the VM. Moreover, the decision relies on parents, so it is important to set realistic expectations as to how treatment can improve the prognosis. Among the possible causes that may tip the scale towards an intervention are the need to control lesion size, pain control, aesthetic impairment, functional restriction, ulceration or thromboembolic events (9).

The choice of treatment is personalized and usually follows a progressive increase in complexity, normally starting off with more conservative alternatives and leaving surgery for selected cases. If possible, a strategy of “wait and see” is preferred. However, there are cases in which it is necessary to intervene, with minimal invasive percutaneous sclerotherapy being the treatment of choice for VMs. Surgery is preferred when there is an evident compromise of the airway, size limitation, important aesthetic impairment or pain, among others. Laser is an alternative adjuvant therapy to treat aesthetic defects. Any option requires of a multidisciplinary team, as often there is a follow up period needed, multiple approaches might be combined or the patient may present another vascular malformation associated (11)(12). Recently, genetic testing has allowed to identify mutations which can be targeted with drugs and allow for less invasive approaches, setting up an interesting field of research (13).

To summarize, we would like to enhance the importance of making a correct anamnesis and physical examination upon the finding of a soft tissue tumor on a pediatric patient. It is crucial to avoid assuming that a mass proximal to the intraoral region has necessarily an odontogenic origin, as there are other clinical possibilities. In light of this, performing an echography upon the suspicion of a VM is extremely useful as it might avoid serious complications from undergoing more aggressive procedures. One does not diagnose what one does not have in mind, and although they are rare, vascular malformations should be included in the differential diagnosis of a tumor in the head and neck region.
